# Effect of C-Clamp Application on Hemodynamic Instability in Polytrauma Victims with Pelvic Fracture

**DOI:** 10.3390/medicina58091291

**Published:** 2022-09-16

**Authors:** Jan Gewiess, Markus Martin Luedi, Beat Schnüriger, Theodoros Hercules Tosounidis, Marius Johann Baptist Keel, Johannes Dominik Bastian

**Affiliations:** 1 Department of Orthopaedic Surgery and Traumatology, Inselspital, Bern University Hospital, University of Bern, 3010 Bern, Switzerland; 2 Department of Anaesthesiology and Pain Medicine, Inselspital, Bern University Hospital, University of Bern, 3010 Bern, Switzerland; 3 Department of Visceral Surgery and Medicine, Acute Care Surgery Team, Inselspital, Bern University Hospital, University of Bern, 3010 Bern, Switzerland; 4 Department of Orthopaedic Surgery, University Hospital Heraklion Crete, 71500 Heraklion, Crete, Greece

**Keywords:** pelvic C-clamp, hemodynamic instability, pelvic ring fracture, emergency management

## Abstract

*Background and Objectives:* C-clamp application may reduce mortality in patients with unstable pelvic fractures and hemodynamic instability. Decreasing C-clamp use over the past decades may have resulted from concerns about its effectiveness and safety. The purpose of this study was to document effective hemodynamic stabilization after C-clamp application by means of vital parameters (primary outcome parameter), and the subsequent effect on metabolic indices and volume management (secondary outcome parameters). *Materials and Methods:* C-clamp application was performed between 2014 and 2021 for *n* = 13 patients (50 ± 18 years) with unstable pelvic fractures and hemodynamic instability. Vital parameters, metabolic indices, volume management, and the correlation of factors and potential changes were analyzed. *Results*: After C-clamp application, increases were measured in systolic blood pressure (+15 mmHg; *p* = 0.0284) and mean arterial pressure (+12 mmHg; *p* = 0.0157), and a reduction of volume requirements (*p* = 0.0266) and bolus vasoactive medication needs (*p* = 0.0081) were observed. The earlier C-clamp application was performed, the greater the effect (*p* < 0.05; r > 0.6). Heart rate, shock index, and end-tidal CO_2_ were not significantly altered. The extent of base deficit, hemoglobin, and lactate did not correlate with changes in vital parameters. *Conclusions*: In the majority of hemodynamically unstable trauma patients not responding to initial fluid resuscitation and severe pelvic fracture, early C-clamp application had an additive effect on hemodynamic stabilization and reduction in volume substitution. Based on these findings, there is still a rationale for considering early C-clamp stabilization in this group of severely injured patients.

## 1. Introduction

Ever since its clinical introduction in 1989 by Reinhold Ganz, the pelvic C-clamp has been used in the emergency treatment of patients with unstable posterior pelvic ring injuries and hemodynamic instability to prevent excessive blood loss, which is present in 1–2% of all pelvic fractures [1,2,3,4,5]. However, despite initial enthusiasm, some complications, uncertainties over its effectiveness, and the routine use of pelvic binders in the prehospital phase have led to reservations with regards to C-clamp application [6,7]. 

In trauma patients, uncontrolled bleeding is the most important cause of early and potentially preventable death [8]. Lesions of the posterior pelvic ring have an 80% risk of concomitant and potentially fatal hemorrhage, with reported mortality rates of up to 57% [3,9,10,11,12]. In these cases, relevant and potentially occult pelvic bleeding originates from the venous plexus or cancellous bone in 80–90% of patients [11,13,14]. The discontinuity of the pelvic compartment borders caused by damage to ligaments of the pelvic ring, pelvic floor and iliopectineal fascia results in an increased pelvic volume and limits self-tamponade in a ‘chimney effect’ [2,15,16].

Fluid resuscitation remains the indisputable mainstay in the treatment of hemodynamic shock and may be combined with vasopressor administration [17,18,19,20]. Hemorrhage control is achieved by either direct compression, the interruption of the blood supply proximal to the bleeding site (i.e., angioembolization, resuscitative endovascular balloon occlusion of the aorta (REBOA), aortic clamp) or local tamponade distal to the bleeding site (i.e., pelvic binder, pelvic C-clamp, pelvic packing). In this context, there is a consensus about the effective closing of bleeding surfaces, tamponade, the prevention of vessel shear and allowance of clot maturation under early pelvic binder or C-clamp stabilization, with the latter permitting the unrestricted performance of all relevant abdominal and vascular procedures [2,3,21,22,23,24,25]. However, to the best of our knowledge, there are no prospective randomized trials evaluating the hemodynamic impact of different means of external mechanical pelvic stabilization (e.g., pelvic binder versus C-clamp) and no substantiating evidence for C-clamp use exceeding a grade IIa recommendation [26]. Hemodynamic stabilization as achieved through effective hemorrhage control may be indicated by the stabilization of (i) vital parameters, such as systolic arterial blood pressure (sABP), mean arterial blood pressure (MAP), heart rate (HR), shock index (SI), and end tidal CO_2_ per respiratory minute volume (etCO_2_/RMV), (ii) metabolic indices, such as base deficit, hemoglobin, and lactate, and (iii) volume substitution needs [3,27,28,29,30]. 

We hypothesized, that in hemodynamically unstable blunt trauma patients with unstable pelvic fractures not responding to initial fluid resuscitation, additive C-clamp application contributes to hemodynamic stabilization.

## 2. Materials and Methods

### 2.1. Inclusion and Exclusion Criteria

Adult trauma patients admitted to the emergency department of a level I trauma center between 2014 and 2021 with posterior pelvic ring disruption with hemodynamic instability and pelvic C-clamp application were included. Hemodynamic instability was assumed if any of the following were present within 30 min before C-clamp application [27]: systolic arterial blood pressure (sABP) < 90 mmHg, heart rate (HR) > 100 min^−1^, mean arterial blood pressure (MAP) < 70 mmHg, or shock index (SI) > 1. Patients were excluded if hemodynamic stabilization was achieved >30 min prior to C-clamp application, if C-clamp application was temporarily performed during definitive operative stabilization only or if there were incomplete medical records. Patients with associated injuries were not excluded. 

### 2.2. Assessment of Hemodynamic Stabilization

The hemodynamic state was assessed by means of (i) vital parameters (sABP, MAP, HR, SI, etCO2/RMV), (ii) metabolic indices (base deficit (BD), hemoglobin, lactate), (iii) volume management (packed red blood cells (PRBC), fresh frozen plasma (FFP), platelet concentrate (PC), crystalloid solution (NaCl and Ringer’s lactate), requirement for and dosage for continuous and bolus vasoactive medication). The stabilization of vital parameters was considered to reflect hemodynamic stabilization and prevent the development of shock [27,29] and was thus defined as the primary outcome parameter. Subsequent courses of volume replacement and metabolic indices indicating the severity or persistence of hemorrhage [28,31] and vasopressor use reflecting inadequate resuscitation and mortality risk [32,33] were defined as secondary outcome parameters. 

The effect of the pelvic C-clamp was assessed in two timeframes as follows (1) within 30 min before and 30 min after C-clamp application for vital parameters and (2) from the time of arrival at the emergency department to C-clamp application and the first hour after C-clamp application for volume and vasopressor infusion rates. Factors related to effective vital parameter stabilization were investigated. Courses of metabolic indices were followed for 12 h and the time from C-clamp application until normalization was assessed. Both, the initial excess of indices and time until normalization were correlated to changes in vital parameters after C-clamp application. 

### 2.3. Statistical Analysis

Statistical analyses were performed using R (R Foundation for Statistical Computing, Version 4.1.0 (2021), Vienna, Austria) and GraphPad Prism (GraphPad Software, Version 9.3.1 (2022), La Jolla, San Diego, CA, USA). Descriptive statistics are displayed using mean and standard deviation (SD). The normal distribution was detected using the Shapiro-Wilk test. Group comparisons were performed using *t*-tests and Mann-Whitney *U* tests, and paired *t*-tests and Wilcoxon signed-rank tests. Correlation analyses were performed using Pearson’s and Spearman’s methods. The level of significance was set at *p* = 0.05. No *p*-value adjustment for multiple comparisons was performed as the analysis was pre-defined. 

## 3. Results

### 3.1. Patients

**Figure 1:**
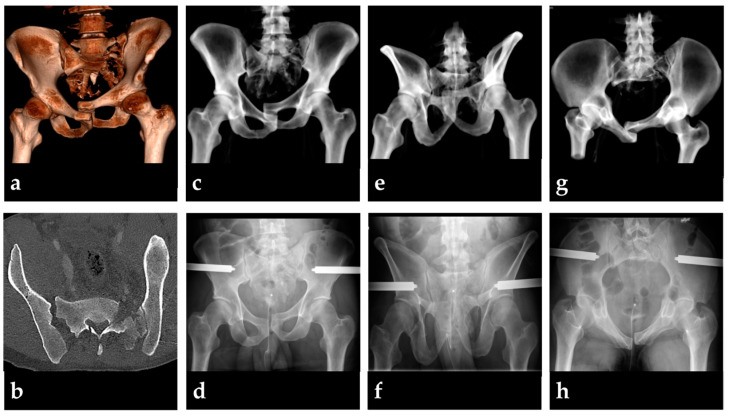
Example of an unstable pelvic fracture in a 40-year-old patient presenting at the emergency department with hemodynamic instability after falling from a 15 m height (ISS = 50, GCS = 13, initial lactate 5.8 mmol/L). A pelvic binder was placed in the prehospital phase and resulted in overreduction. A 3D reconstruction of the initial CT scans with the binder in place reveals the vertical shear pelvic injury pattern (**a**), with gross posterior instability resulting from bilateral transforaminal sacral fracture and unilateral sacroiliac dislocation (**b**). Initial fracture reduction and application were successfully performed using a pelvic C-clamp as shown in a.p., outlet, and inlet projections before (**c**,**e**,**g**) and after application (**d**,**f**,**h**). Application resulted in significant increases of sABP and MAP and a decrease in HR and SI, respectively (*p* < 0.05).

Between 2014 and 2021, 20 patients received a pelvic C-clamp during the initial treatment of unstable pelvic ring fractures (Figure 1). Study inclusion criteria were met by 13 patients (50 ± 18 years; *n* female = 4; *n* male = 9; Table 1). Seven patients were excluded because of hemodynamic stabilization before C-clamp stabilization (respective mean values of sABP > 90 mmHg, HR < 100 min-1, MAP > 70 mmHg, and SI < 1 during 30 min before C-clamp stabilization). The mean injury severity score (ISS) was 45 ± 14. In ten patients (77%), a pelvic binder was in place on admission, with sufficient placement being assumed in nine patients. In six patients with a pelvic binder, a concomitant acetabular fracture, proximal femur fracture, or hip dislocation was present. Other treatments prior to C-clamp application consisted of resuscitative thoracotomy (*n =* 1), and REBOA (*n* = 2). Treatments following C-clamp application consisted of emergency laparotomy (*n* = 4) and embolization (*n =* 2). Within the study period, no complications associated with the pelvic C-clamp were observed. Death occurred in three patients (23%). One patient died six hours after admission; a second patient died two days after admission, and the third patient died 20 days after admission, presumably due to massive pulmonary embolism. 

### 3.2. Vital Parameters

**Figure 2:**
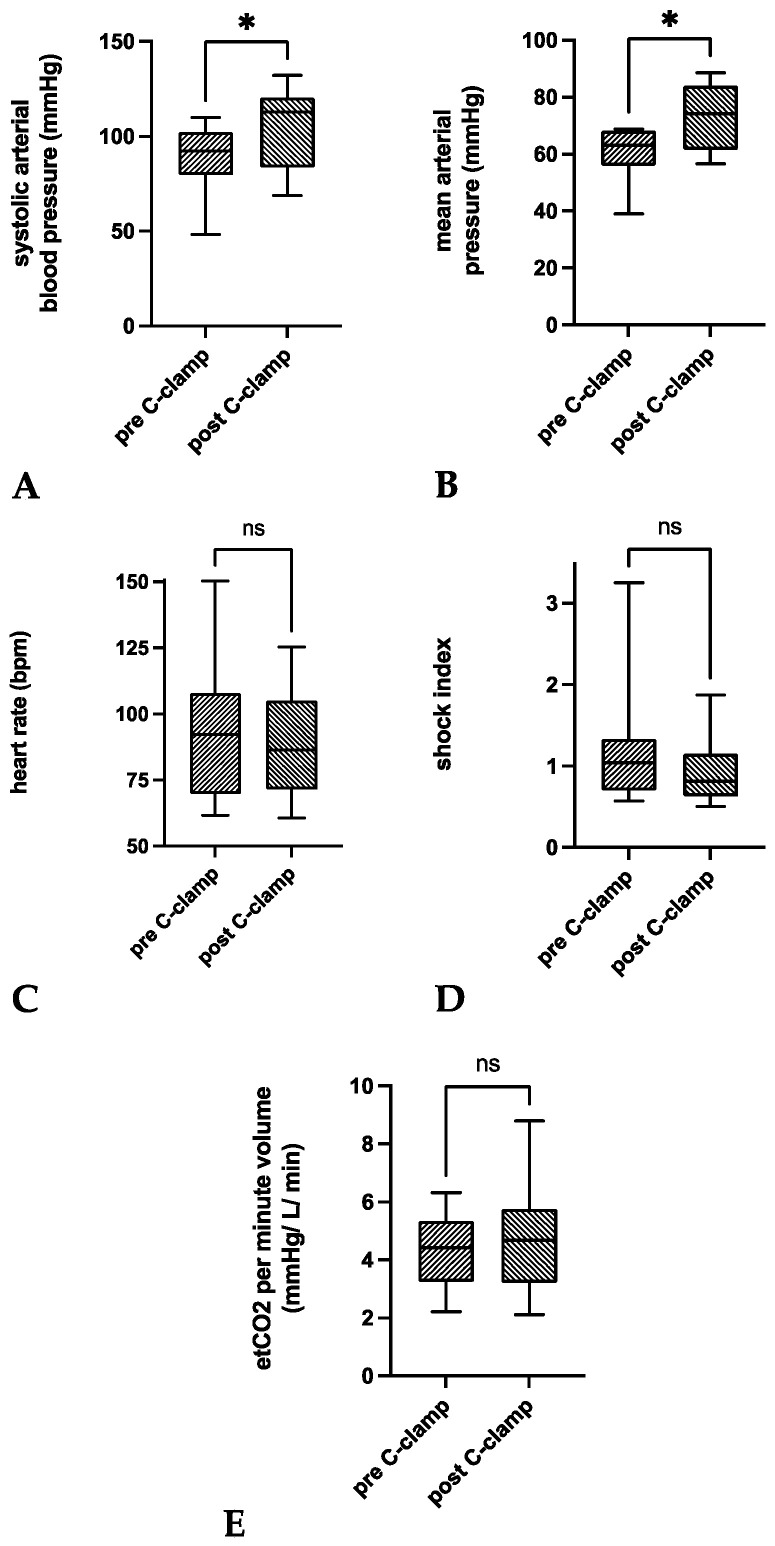
Box plots of the pairwise comparisons of means 30 min before (pre C-clamp) and after (after C-clamp) C-clamp application for (**A**) sABP, (**B**) MAP, (**C**) heart rate, (**D**) shock index, and (**E**) etCO_2_ per minute volume. Paired comparisons of respective means revealed a significant mean increase of sABP (+14.9 mmHg; 95% CI 1.9–28; *p* = 0.0284) and MAP (+12.1 mmHg; 95% CI 2.1–23.5; *p* = 0.0157) after C-clamp application. Mean HR, SI, and etCO_2_ per minute volume did not significantly differ before and after C-clamp application. Box—interquartile range; bar—median. * *p* < 0.05.

Compared to 30 min before C-clamp application, a significant increase of mean sABP by +14.9 ± 21.6 mmHg was observed within 30 min after C-clamp application (*p* = 0.0284; Table 2, Figure 2). 

The acute hemodynamic stabilization of sABP >90 mmHg was achieved in *n =* 8 patients (62%). There was a significant increase of mean MAP by +12.1 ± 15.5 mmHg (*p* = 0.0157). MAP was stabilized to values >70 mmHg in *n =* 8 patients (62%). Mean HR decreased by Ȓ6.1 ± 11.1 min^−1^ (*p* = 0.0708). A decrease of HR was observed in *n =* 10 patients (77%). A decrease <100 min^−1^ was present in *n =* 2 patients (15%). Mean SI decreased by −0.3 ± 0.6 (*p* = 0.0971). A decrease was observed in *n =* 10 patients (77%). SI stabilization < 1 was achieved in *n =* 3 patients (23%). Mean etCO_2_ minute volume ratio did not significantly improve (*p* = 0.3082). 

**Figure 3:**
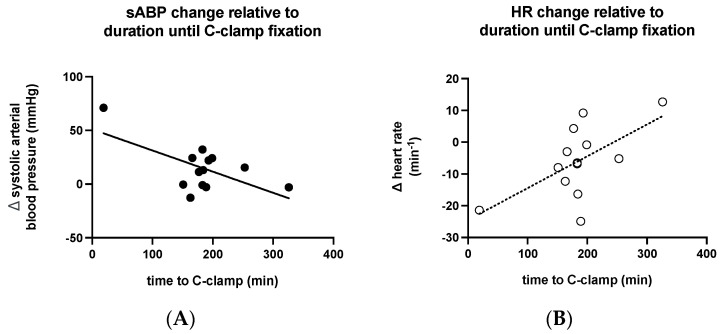
Relation of the magnitude of the effect on (**A**) sABP and (**B**) HR and time passed until C-clamp application. The earlier C-clamp application was performed, the greater the effect regarding sABP elevation. The earlier C-clamp application was performed, the greater the effect of HR reduction.

A shorter time to C-clamp application was associated with a greater increase in the mean sABP (*p* = 0.0253, r = -0.6149; Figure 3A), a greater decrease in HR (*p* = 0.027, r = 0.6094; Figure 3B), and a greater decrease in SI (*p* = 0.0012, r = 0.7929). Age, weight, admission GCS, initial lactate, initial hemoglobin, initial pH, ISS, and the estimated extent of blood loss were not significantly correlated with changes in vital parameters. 

### 3.3. Metabolic Indices and Volume Management

Mean initial BD, hemoglobin, and lactate were out of normal range (Table 3). The extent of initial excess was not significantly correlated to changes in vital parameters with C-clamp application. The mean time until normalization of respective values was more than twelve hours in most patients and did not permit proper statistical analyses. 

**Figure 4:**
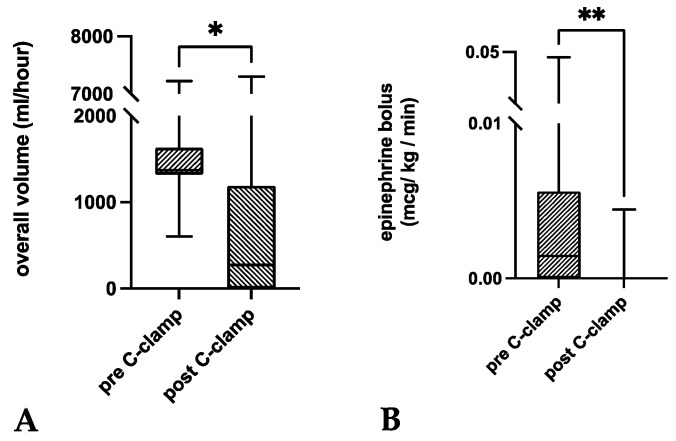
Box plots of the pairwise comparisons of medians before and after C-clamp application for the (**A**) overall volume administered per hour and the (**B**) epinephrine bolus. Both the volume substitution and epinephrine bolus administration significantly decreased within the first hour after C-clamp application (*p* < 0.05). Box—interquartile range; bar—median. * *p* < 0.05; ** *p* < 0.01.

Compared to time intervals from admission until C-clamp application, volume substitution infusion rates were significantly lower within one hour after C-clamp application (*p* = 0.0266; Figure 4). This was especially the case in infusion rates of Ringer’s lactate solution (*p* = 0.0081). There was no significant decrease in PRBC, FFP, PC, and NaCl transfusion rates within the first hour after C-clamp application (*p* > 0.05). 

Within one hour of C-clamp application, significantly lower infusion rates of a norepinephrine bolus were administered compared to time intervals from admission until C-clamp application (*p* = 0.0039; Table 3 and Figure 4). Both continuous vasopressor administration and bolus administration were necessary in all patients. There was no significant difference regarding the flow rate of norepinephrine (*p* = 0.0645).

## 4. Discussion

The decrease in C-clamp use over the past decades may be a result of concerns about the correct indication, technique, effectiveness, and safety of this procedure [7]. Despite great variation (8% to 58%) in reported mortality rates after C-clamp application [2,3,6,14,15,20,25,34,35,36,37]), it is believed to contribute to a reduction in mortality [OR 0.68 (0.49–0.95)] [6]. Therefore, many authors favor the use of pelvic C-clamp application before extra- and/or intraperitoneal pelvic packing and angiography [11,38,39,40,41]. Interestingly, although C-clamp application was shown to exert considerably higher posterior compression forces of up to 685 N compared to other means of external fixation [21,22,41,42], only 46% of orthopedic surgeons would confidently use a pelvic C-clamp [43]. To overcome this uncertainty, several authors advocate the implementation of training sessions, which have been shown to result in pin placement in the safe area within 6 min in 90% of cases [23,43,44].

Pelvic binders are often considered advisable if not superior to C-clamp application, as the procedure is simple, cost-effective, non-invasive, and timesaving [37]. However, pelvic binders facilitate not more than 25% of the compressive forces of the C-clamp at the sacroiliac level [45]. Furthermore, pelvic binders are supposed to be an integral part of the preclinical phase, similar to routine C-spine immobilization for suspected cervical trauma [7,37,40,45,46,47]. However, application may not be effective in patients with vertical shear type fractures, concomitant proximal femur, or acetabular fractures (60% in the present study and 1.5% of pelvic fractures requiring emergency external stabilization [7]) and carries a risk of overreduction, misplacement, and pressure sores after 24–36 h [45,48,49,50]. In the context of the interregional variation of preferences and algorithms [14,26,40,51], the clarification of the resuscitative potential of C-clamps within the cascade of emergency management is essential. 

In Tile types B and C, Young and Burgess types APCII/III, and VS fractures [2,3,6,7,14,15,20,28,35,37,52,53,54,55], pelvic C-clamp application has long been a component of clinical practice, aimed at achieving direct sacroiliac compression for hemorrhage control (‘first line of defense’ [11]) [2,3,21,34,38]. Additionally, C-clamp application can be used instead of pelvic binders if a patient’s condition does not permit timely, definitive stabilization or to facilitate nursing and transfer. The extent of radiologic fracture dislocation is an impractical parameter deciding for or against pelvic C-clamp application given the widespread use of pelvic binders in the prehospital phase. Similarly, fracture classifications discount the extent and severity of extra- and intrapelvic as well as neurovascular lesions [38,53]. Thus, deduction of therapeutic consequences should rely on a more extensive approach including the grade of hemodynamic instability [36]. Defining and grading hemodynamic instability in connection with hemorrhage remains a challenge [11,27,56]. Reports on the effects of pelvic C-clamp application on hemodynamic parameters are rare and vague in terms of quantitative and temporal relations [7,15,35,53].

For the assessment of the stabilization of vital parameters, we evaluated sABP, MAP, HR, SI and the etCO_2_/RMV ratio. Our results indicate a significant contribution of C-clamp application to the stabilization of vital parameters. Earlier studies elaborating on the hemodynamic effect of pelvic C-clamps reported hemodynamic instability as follows: (i) ‘hypovolemic shock’ [52], (ii) estimated blood loss >1500 mL (ATLS^®^ class III-IV hemorrhage) [2,52], (iii) HR >100 min^−1^ [52], (iv) sABP <90–100 mmHg (despite 2 L fluid administration) [20,35,36,37,52], (v) SI > 1 [3], (vi) delayed capillary refill [52]), and (vii) not further specified [15,28]. Hemodynamic improvement was observed in seven studies with sample sizes ranging from eleven [15] to 40 patients [37]: for vital parameters, the stabilization of sABP was reported in five studies [20,35,36,37,52], and the stabilization of MAP was observed in one study [15]. 

In blunt trauma patients, sABP is the most commonly used parameter to define hemodynamic instability [27]. The stabilization of sABP has been shown to effectively reduce mortality [12,57]. Considering the balancing act of sABP correction in hemorrhage (maintenance of end organ perfusion versus allowance for clot maturation), the attainment of cut-off values within a given range seems more important compared to the absolute sABP increase [27,58]. While sABP is known to be rather insensitive with regard to the detection of hemorrhage because it changes with levels of pain, emotional distress, and hypoxia [14,20,31], underlying mechanistic considerations can only partially be assumed for the stabilization of hemorrhage. The contribution of pelvic C-clamp application to sABP stabilization in 62% of patients that did not respond to initial fluid resuscitation is the most important finding of this retrospective study. To the best of our knowledge, this has neither been previously reported with regard to time intervals allowing a potential minimization of complementary or simultaneous interventions, nor in relation to respective values before C-clamp application: Richard et al. observed a mean sABP elevation of 23 mmHg in an unclear ratio of eleven patients at 15 min after C-clamp application, compared to values at the time of clamp tightening [25]. Others used sABP for the characterization of hemodynamic stability but did not report on its course after C-clamp application [20,35,36,37,52]. 

Maintenance of MAP > 60 mmHg is important for regular end organ perfusion. MAP reportedly is associated with mortality whereas significantly lower initial MAP was reported in non-survivors compared to survivors [14,59]. Similar to sABP interpretation, in MAP, the correction into a specific range seems more important compared to the absolute increase itself [58]. Our results indicate an important contribution of the pelvic C-clamp application to MAP stabilization. In line with these findings, Tiemann et al. observed an increase in MAP by a mean of 25% at 20 min after C-clamp application [15]. The consolidation of MAP to values of >70 mmHg was observed at six hours after C-clamp application. In contrast to our study, this effect was only present in surviving patients, and MAP stabilization was not observed in non-survivors. 

Although HR is often used to describe hemodynamic instability, its informative value remains questionable [27]. SI as a function of HR underlies similar preconditions. The etCO_2_/RMV ratio permits the estimation of global cardiopulmonary function during resuscitation, as it is an estimate of cardiac output and organ perfusion [30]. It has been considered a useful monitoring tool during resuscitation in cardiac arrest [60]. HR, SI, and etCO_2_/RMV did not stabilize significantly after C-clamp application. However, some sort of HR and SI stabilizing trend must be assumed, given the consistent effect in 77% of patients. To the best of our knowledge, changes in these parameters after C-clamp application have not yet been reported. 

For sABP, HR, and SI, the respective stabilizing effect was greater with less time until application. The most plausible explanation includes the effect of preceding interventions or mechanisms given the underlying injury severity (e.g., other surgical interventions for bleeding control, open cardiac massage, REBOA, chest tube for tension pneumothorax, fluid administration, clot maturation). In this context, mean ISS (45) and mean time until C-clamp application (184 min) were considerably higher in the present study compared to literature findings (mean ISS = 20 to 42 [3,7,14,15,20,35,36,37,53,55] and mean time until C-clamp = 12 to 110 min [2,3,7,14,15,20,35,37]). Theoretically, C-clamp application may be delayed by preceding diagnostic and therapeutic interventions and transport times or capacities when application is performed in the operating room. Given the ratio of patients admitted with pelvic binders (10/13), the thorough evaluation of CT scans and estimation of the additional benefit of pelvic C-clamp stabilization against early definitive stabilization is another important explanation for the observed delay. 

For the assessment of the stabilization of metabolic indices, we evaluated blood lactate, base deficit, and hemoglobin. Our results do not indicate a direct contribution of C-clamp application to the stabilization of metabolic indices. Increasing blood lactate and base deficit indicate hypovolemia-induced tissue hypoperfusion and the severity of bleeding and are associated with poor survival [14,20,28]. In addition, the persistence of elevated lactate levels has been suggested to be an indicator of ongoing hemorrhage [14]. Hemoglobin has been shown to indicate hemorrhage within minutes and can be used for an estimation of blood loss in trauma patients [2,61]. Initial BD, hemoglobin, and lactate levels indicate severe hemorrhage in most of the patients in this study. However, the short-term normalization of metabolic indices after pelvic C-clamp rarely occurred. In most patients, normalization was not achieved within twelve hours. In line with our results, initial hemoglobin is considered a poor indicator for predicting survival, and may conceal cellular perfusion and tissue oxygenation deficits [20]. The retrospective evaluation of C-clamp effectiveness based on BD, hemoglobin, and lactate levels is difficult because it is complicated by the adaption of initial and subsequent treatment concepts to momentary and undulant courses of metabolic indices taken at incoherent points in time. 

For the assessment of volume substitution needs we evaluated the infusion rate of packed red blood cells (PRBC), fresh frozen plasma (FFP), platelet concentrate (PC), and crystalloid solution (NaCl and Ringer’s lactate). Our results indicate a significant contribution of C-clamp application to a reduction of volume substitution needs. A reduction in the need for volume substitution indicates the discontinuation of hemorrhage and has been interpreted as an expression of hemodynamic stabilization [3]. Non-survivors reportedly require greater amounts of volume substitution compared to survivors [14]. An interesting finding in the present study is the significant reduction of volume substitution needs after C-clamp application. However, the inclusion of the initial bolus administration in accordance with ATLS^®^ guidelines in time intervals before C-clamp application may confound our results. In the hemodynamically unstable patient requiring C-clamp application, reported average quantities of blood products given ranged from 17 to 38 PRBC within the first twelve hours [3,20,35,53] and from twelve to 18 within the first three hours [2]. In line with our findings, Heini et al. reported an ‘improvement as seen by a reduction of fluid infusion and/or a reduction of the pulse/blood pressure ratio’ in ten out of 18 patients (56%) [3], and Tiemann et al. reported a decrease in blood product administration at six hours after C-clamp application [15]. Without specifying the underlying timeframe, Sadri et al. did not report a decrease in the number of transfused blood products in 14 patients before and after C-clamp application [35]. Considering complications secondary to massive transfusion or crystalloid fluid administration (e.g., coagulopathy [62]), C-clamp application may provide a fluid-sparing approach. 

While vasoactive medication is an integral part of the treatment of septic shock, its use is controversial in trauma patients [32,63,64]. In hemorrhagic shock, vasopressive medication should be terminated as soon as possible upon the establishment of normovolemia due to a potential association with increased mortality [33]. The course of vasoactive medication has not yet been reported to be a parameter for consideration in the evaluation of hemodynamic stabilization after C-clamp application. Our results demonstrate a contribution of C-clamp application to a significant decrease of vasopressor bolus needs while the infusion rates of continuous vasoactive medication were not significantly altered. This may be explained by the correction of sABP into rather low normal ranges, indicating a persistent need for vasopressor support, while hypotensive crises were rarely observed, and bolus administration was no longer necessary after C-clamp application.

The missing response of singular parameters in some patients may be secondary to ongoing hemorrhage requiring further means of resuscitation. Furthermore, the loss of reduction and the loosening of the C-clamp may impede its effectiveness. Such complications have been reported in 8–13% of patients [3,25]. Pin malpositioning and migration were observed in 3–5% [2,3]. Other reasons may include pin perforation, overreduction, or underreduction. 

The limitations of the study are (i) the low number of included patients, (ii) a missing control group, (iii) the retrospective study design without randomization for treatment, and (iv) the attempts to attribute effects from the application of the C-clamp as a single intervention. The small sample size can be explained by the rarity of a pelvic injury requiring C-clamp fixation as an emergency procedure for hemodynamic instability, a routine use of pelvic binders in the prehospital phase, and a trend towards early definitive stabilization. Furthermore, the rarity of the injury results in long observation periods to collect data. In addition, the heterogeneity of sustained trauma, relevant comorbidities possibly alternating bleeding response, and resuscitative interventions, as well as the necessity for simultaneous interventions in often combined and complex injuries results in the inability to make comparisons of cases. Thus, any positive effect of the pelvic C-clamp outlined in the present study must be considered additive at best. Furthermore, ethical considerations hinder comparisons with control populations in general and in a setup of a prospective randomized clinical trial in particular. The strength of the presented study is that we reported on hemodynamic stabilization after C-clamp application comprehensively for the first time.

## 5. Conclusions

In the majority of hemodynamic unstable blunt trauma patients not responding to initial fluid resuscitation, early pelvic C-clamp application had an additive effect on the stabilization of vital parameters and the reduction of volume substitution needs. For a further evaluation of its performance in the face of different emergency interventions regarding improved patients’ selection, fracture reduction and hemodynamic stabilization, randomized controlled trials would be desirable, yet are probably not feasible because of ethical considerations.

## Figures and Tables

**Table 1:** Age, gender, injury severity score, trauma mechanism, and fracture classification in patients with unstable pelvic ring fractures with hemodynamic instability and treated by C-clamp application.

ID	Age	Gender	ISS	Trauma Mechanism	Young/Burgess Type
1	31	m	54	fall from height	VC
2	51	f	38	MVA	VC
3	27	m	38	fall from height	VC
4	28	m	50	fall from height	VC
5	53	m	33	fall from height	VC
6	39	f	29	MVA	APCIII
7	42	m	45	other	LCIII
8 †	69	m	38	fall from height	VC
9	60	f	43	MVA	LCIII
10 †	64	m	75	MVA	APCIII
11	40	m	50	fall from height	VC
12	74	m	66	MVA	VC
13 †	74	f	29	MVA	APCII
mean ± SD	50.2 ± 17		45.2 ± 13.8		

APC = anteroposterior compression; f = female; ISS = injury severity score; m = male; MVA = motor vehicle accident; SD = standard deviation; VC = vertical shear. † non-survivors.

**Table 2:** Primary outcome parameters from 30 min before to 30 min after C-clamp application in patients with unstable pelvic ring fractures and hemodynamic instability.

ID	ΔsABP (mmHg)	ΔMAP (mmHg)	ΔHR (min^−1^)	ΔSI	ΔetCO_2_/RMV (mmHg/L/min)
1	−13	−11	−12	0.0	−0.2
2	32	34	−7	−0.4	0.0
3	71	44	−21	−2.2	3.0
4	24	15	−3	−0.4	−0.5
5	11	8	4	0.0	3.5
6	−1	0	−8	−0.1	2.0
7	13	19	−16	−0.2	−1.0
8 †	22	17	9	0.0	−0.6
9	−1	−2	−7	0.0	−0.1
10 †	−3	−2	−25	−0.2	−0.2
11	15	12	−5	−0.2	0.3
12	−3	1	13	0.2	−0.1
13 †	24	23	−1	−0.2	−0.5

etCO2/RMV = end tidal CO_2_ per minute volume; HR = heart rate; MAP = mean arterial pressure; sABP = systolic arterial blood pressure; † non-survivors.

**Table 3:** Secondary outcome parameters before and after C-clamp application in patients with unstable pelvic ring fractures and hemodynamic instability.

ID	Initial BE (mmol/L)	Time until Normalization (hh:mm)	Initial Hemoglobine (g/L)	Time until Normalization (hh:mm)	Initial Lactate (mmol/L)	Time until Normalization (hh:mm)	VS before C-Clamp (mL/h)	VS after C-Clamp (mL/h)
1	−5.7	>12 h	70	>12	4.6	06:15	1690	1275
2	−7.6	>12	113	>12	2.7	>12	1450	0
3	−20.5	>12	91	>12	12.3	11:59	846	5150
4	−8.6	03:37	114	>12	5.3	>12	1317	250
5	−5.7	00:01	130	>12	4.4	00:01	1371	0
6	−1.8	08:35	127	>12	2.5	>12	1757	0
7	−3.4	>12	136	>12	1.1	n.a.	604	0
8 †	−11.4	>12	117	01:52	7.2	01:52	1321	750
9	−1.9	>12	126	>12	1.8	n.a.	1513	275
10 †	−20.4	>12	106	>12	13.4	>12	7224	7300
11	−10.5	00:14	99	>12	5.8	00:14	1340	1100
12	−7.6	>12	107	>12	8.1	>12	1368	300
13 †	−9.2	>12	109	>12	1.8	00:59	1573	0
mean ± SD	−8.8 ± 6		111.2 ± 17.8		5.5 ± 3.9		1798 ± 1595	1262 ± 2287

BE = base excess; n.a. = not applicable; VS = volume substitution needs; normal ranges: BE −2.5 ± 2.3 mmol/L; hemoglobin 135–168 g/L; lactate 0.63–2.44 mmol/L; † non-survivors.

## Data Availability

The data presented in this study are available on request from the corresponding author. The data are not publicly available due to privacy reasons.
